# Cross-sectional associations between domain-specific sitting time and other lifestyle health behaviours: the Stormont study

**DOI:** 10.1093/pubmed/fdab298

**Published:** 2021-08-03

**Authors:** Victoria E Kettle, Mark Hamer, Fehmidah Munir, Jonathan Houdmont, Kelly Wilson, Robert Kerr, Ken Addley, Lauren B Sherar, Stacy A Clemes

**Affiliations:** National Centre for Sport and Exercise Medicine, School of Sport, Exercise and Health Sciences, Loughborough University, Loughborough LE11 3TU, UK; Institute of Sport, Exercise and Health, UCL, London WC1E 6BT, UK; National Centre for Sport and Exercise Medicine, School of Sport, Exercise and Health Sciences, Loughborough University, Loughborough LE11 3TU, UK; Faculty of Medicine and Health Sciences, University of Nottingham, Nottingham NG7 2RD, UK; Department of Management and Leadership, Ulster University, County Antrim BT37 0QB, UK; Department of Management and Leadership, Ulster University, County Antrim BT37 0QB, UK; Faculty of Occupational Medicine, Royal College of Physicians of Ireland, Dublin D02 X266, Ireland; National Centre for Sport and Exercise Medicine, School of Sport, Exercise and Health Sciences, Loughborough University, Loughborough LE11 3TU, UK; National Centre for Sport and Exercise Medicine, School of Sport, Exercise and Health Sciences, Loughborough University, Loughborough LE11 3TU, UK; National Institute for Health Research (NIHR), Leicester Biomedical Research Centre, University Hospitals of Leicester NHS Trust and the University of Leicester, Leicester LE5 4PW, UK

**Keywords:** alcohol drinking, diet, exercise, food, nutrition, sedentary behaviour, smoking, workplace

## Abstract

**Background:**

There is a dearth of literature on how different domains of sitting time relate to other health behaviours. Therefore, this study aimed to explore these associations in a sample of office workers.

**Methods:**

7170 Northern Irish Civil Servants completed an online survey which included information on workday and non-workday sitting time in five domains (travel, work, TV, computer-use, leisure-time), physical activity, fruit and vegetable intake, alcohol consumption and cigarette smoking. An unhealthy behaviour score was calculated by summing the number of health behaviours which did not meet the current guidelines. Multinomial regressions examined associations between unhealthy behaviour score and each domain of sitting time.

**Results:**

≥7 hours sitting at work and ≥2 hours TV viewing on a workday both more than doubled the odds of partaking in ≥3 unhealthy behaviours [Odds ratio, OR = 2.03, 95% CI, (1.59–2.61); OR = 2.19 (1.71–2.80)] and ≥3 hours of TV viewing on a non-workday nearly tripled the odds [OR = 2.96 (2.32–3.77)].

**Conclusions:**

High sitting time at work and TV viewing on a workday and non-workday are associated with increased odds of partaking in multiple unhealthy behaviours. Interventions need to focus on these domains and public health policy should consider sitting time as an important health behaviour.

## Introduction

The negative health consequences of cigarette smoking, fruit and vegetable underconsumption, physical inactivity and alcohol overconsumption are well established.^1–4^ Additionally, sedentary behaviour defined as, ‘any waking behaviour characterized by an energy expenditure ≤ 1.5 metabolic equivalents (METs), while in a sitting, reclining or lying posture’,^5^ is associated with numerous chronic diseases and increasingly prevalent.^6^^,^^7^ A large European study found that on average, adults spent 530 minutes/day sedentary.^8^ Due to the emergence of sedentary behaviour and evidence that health behaviours typically coexist,^9^ it is necessary to explore the associations between sedentary behaviour and other health behaviours.

Previous studies measuring sitting time as a proxy for sedentary behaviour have shown that certain sitting time domains are associated with other health behaviours.^10–12^ A review exploring all measures of sedentary behaviour (TV viewing, total sitting time, general screen time, occupational and total sedentary time) found an inverse association with physical activity.^13^ Conflicting results have been found for TV viewing and smoking with five studies showing a positive association and four reporting no association. Total sitting time had no association with smoking in all five studies reviewed. The relationship between alcohol consumption and sedentary behaviour is also unclear with two studies reporting an inverse association in females but most studies found no relationship with TV viewing or total sitting time.^14^ Conversely, Pearson and colleagues found a consistent inverse association between TV viewing and fruit and/or vegetable consumption.^15^

Partaking in more than one unhealthy behaviour is likely to increase the negative health consequences.^16^ A review exploring the clustering of smoking, nutrition, alcohol and physical inactivity (‘SNAP’) health risk factors found that most studies reported the clustering of alcohol with smoking and half found that all four behaviours clustered.^9^ However, no study has explored the effect of domain-specific sitting time on multiple unhealthy behaviours. Additionally, most studies examine single sitting time domains in relation to health behaviours, typically TV viewing. Thus, little is known about how other domains relate to other health behaviours.^17^ Identifying which sitting time domains are associated with multiple unhealthy behaviours is essential to inform future interventions and reduce the negative health consequences of both sitting time and potentially other associated unhealthy behaviours.

Therefore, this study aimed to explore the associations between domain-specific sitting time and other health behaviours including physical activity, alcohol consumption, cigarette smoking and fruit and vegetable intake in a sample of Northern Irish office workers. The primary objective was to identify whether specific sitting time domains were associated with multiple other unhealthy behaviours. It was hypothesized that domain-specific sitting time would be associated with increased odds of partaking in multiple other unhealthy behaviours.

## Methods

### Participants and procedure

This cross-sectional study used data from the first (2012) and second (2014) waves of The Stormont Study which tracked a large cohort of employees within the Northern Ireland Civil Service. A voluntary response sampling method was used with all employees invited to take part via their occupational email address; 10 437 office workers who provided informed consent completed the survey (2012, *n* = 5235, 20% response rate; 2014, *n* = 5202, 19%).^18^ Where participants had completed both surveys, only the 2012 data were included to maintain an independent sample. Details of the Stormont Study are discussed elsewhere.^18–20^ The study was approved by the Ethics Committee of Ulster University and conducted in accordance with the Helsinki Declaration.

### Measurement of sitting time

The Domain-Specific Sitting Time Questionnaire (DSSTQ)^21^ asked office workers to ‘estimate how many hours you spend on a typical workday and non-workday in the following situations: whilst travelling to and from places, while at work, while watching television, while using a computer at home, in your leisure-time NOT including television (e.g. visiting friends, movies, dining out, etc.)’. Sitting times reported for each domain were provided in hours and minutes on workdays and non-workdays. The ‘at work’ domain refers to workplace sitting on a workday and working at home on a non-workday thus will be termed as such in this paper. For the purposes of this paper, ‘using a computer at home’ will be termed ‘computer-use’. Domains were summed to produce total sitting time on a workday and non-workday. The DSSTQ has been shown to have acceptable levels of reliability (*r* = 0.23–0.84)^21^ and validity (*r* = 0.40).^22^

### Measurement of other health behaviours

Physical activity was self-reported using a single-item measure which asked participants to report the number of days they conducted ≥30 minutes of moderate-to-vigorous physical activity (MVPA) over the past week.^23^ The use of this measurement tool is recommended when determining if respondents are sufficiently active to benefit their health, it has strong validity (*k* = 0.23) and reliability (*r* = 0.72).^23^^,^^24^ Participants were coded as meeting the current (at the time, the study was conducted) UK guidelines^25^ if they reported ≥30 minutes of MVPA on ≥5 days/week. Participants reported how many units of alcohol they typically consume during the week (Monday–Thursday) and over the weekend (Friday–Sunday). The number of week and weekend units was summed, and participants were categorized as meeting UK guidelines if they consumed ≤14 units/week.^26^ Short-term recall measures of alcohol consumption have been shown to provide the most accurate alcohol intake measurement in a population.^27^ Participants reported if they were a current cigarette smoker or non-smoker and if they were the former, they were categorized as unhealthy. This measure of smoking as a health behaviour is the most common and widely reported in epidemiological studies.^9^ Self-reported fruit and vegetable intake per day was summed and categorized as meeting the current World Health Organization guidelines if ≥5 items were reported.^28^ This two-item serving measure has shown a positive correlation with 24-hour dietary recall values (*r* = 0.27) and strong reliability (*r* = 0.70).^29^

### Socio-demographic variables

Office workers reported their sex, age, educational attainment, marital status, work pattern (full- or part-time), salary band, height and weight. BMI was calculated and categorized into normal weight (<25 kg/m^2^), overweight (25–29.9 kg/m^2^) and obese (≥30 kg/m^2^).^30^ Educational attainment was coded into four groups (school level, further education, university degree or higher degree) and marital status into two groups (married/cohabitating or single/divorced/widowed).

### Statistical analyses

Data from the 2012 and 2014 surveys were pooled as the participant characteristics were similar. Participants were excluded if sitting time >18 hours/day, if data were missing from the at work domain or from >2 domains (*n* = 3007). Additionally, participants were excluded if data were missing for height/weight (*n* = 61), MVPA (*n* = 72), alcohol consumption (*n* = 69), smoking status (*n* = 57) or fruit and vegetable intake (*n* = 1). The number of health behaviours (alcohol consumption, smoking status, MVPA and fruit and vegetable intake) that did not meet current guidelines was summed to produce an unhealthy behaviour score (0–4). For the analyses, the highest two categories were condensed due to a very small percentage of the sample scoring 4 (*n* = 196; 2.7%) to produce four categories.

Descriptive statistics stratified by unhealthy behaviour score and domain-specific sitting time were examined and the differences between groups analyzed using chi-square, independent *t*-tests and ANOVAs. Consequently, domain-specific sitting time was split into tertiles based on the 33.3rd and 66.6th percentiles because currently, there are no clinically meaningful cut-points for sitting time in terms of health. Multinomial regression analyses explored the odds of each domain having all possible unhealthy behaviour scores (ref = score of 0) in terms of domain-specific sitting time (ref = low sitting time). BMI, age, sex, marital status, survey year, salary band, work pattern and education were adjusted for in the final regression models. Statistical significance was set at *P* < 0.05 except for *post-hoc* tests where this value was divided by the number of comparisons made. Analyses were conducted using IBM SPSS Statistics 24 for Windows.

## Results

A total of 7170 office workers (68.7%) provided sufficient data with a mean age of 44.5 ± 9.9 years, 55.0% were female, 70.1% were married/cohabitating and 82.4% worked full-time. A score of 2 was the most common unhealthy behaviour score (41.2%) with physical inactivity being the most prevalent unhealthy behaviour (77.6% not meeting guidelines). The most common combination in office workers partaking in 2 unhealthy behaviours was physical inactivity and fruit and vegetable underconsumption (76.4%). On average, office workers reported sitting for 643 ± 160 minutes on a workday and 491 ± 210 minutes on a non-workday. Table 1 shows the sample characteristics stratified by unhealthy behaviour score.

**Table 1:** Sample characteristics stratified by unhealthy behaviour score

		*Number of unhealthy behaviours*				
		*Total (n = 7170)*	*0 (n = 651)*	*1 (n = 2439)*	*2 (n = 2954)*	*≥3 (n = 1126)*
Year of survey^x^	2012	4332	351 (8.1)	1467 (33.9)	1785 (41.2)	729 (16.8)
	2014	2838	300 (10.6)	972 (34.2)	1169 (41.2)	397 (14.0)
Sex^x^	Male	3321	289 (9.0)	954 (29.6)	1313 (40.8)	665 (20.6)
	Female	3849	362 (9.2)	1481 (37.6)	1637 (41.5)	460 (11.7)
Age	mean ± SD (years)	44.5 ± 9.9	45.9 ± 9.8^a^	45.5 ± 9.7^b^	44.1 ± 9.8^abc^	42.7 ± 10.1^abc^
Marital status^x^	Married/cohabitating	5015	450 (9.0)	1765 (35.2)	2103 (41.9)	697 (13.9)
	Single/divorced/widowed	2155	201 (9.4)	668 (31.2)	846 (39.5)	428 (20.0)
Education^x^	School level	1427	124 (8.7)	475 (33.3)	568 (39.8)	260 (18.2)
	Further education	2425	210 (8.7)	754 (31.3)	1022 (42.1)	439 (18.1)
	University degree	1430	117 (8.2)	492 (34.4)	618 (43.2)	203 (14.2)
	Higher degree	1867	196 (10.5)	711 (38.1)	737 (39.5)	223 (11.9)
Salary Band^x^	>£10 000–£15 000	196	20 (10.2)	66 (33.7)	89 (45.4)	21 (10.7)
	>£15 000–£20 000	988	83 (8.4)	272 (27.5)	415 (42.0)	218 (22.1)
	>£20 000–£25 000	2078	173 (8.3)	706 (34.0)	842 (40.5)	357 (17.2)
	>£25 000–£30 000	1671	150 (9.0)	576 (34.5)	695 (41.6)	250 (15.0)
	>£30 000–£35 000	807	89 (11.0)	292 (36.2)	321 (39.8)	105 (13.0)
	>£35 000–£40 000	762	70 (9.2)	283 (37.1)	309 (40.6)	100 (13.1)
	>£40 000	623	62 (10.0)	229 (36.8)	266 (42.7)	66 (10.6)
Work pattern^x^	Full-time	5881	545 (9.3)	1962 (33.4)	2370 (40.3)	1004 (17.1)
	Part-time	1289	100 (8.0)	463 (136.9)	573 (45.7)	119 (9.5)
BMI category^x^	Normal weight	2561	268 (10.5)	935 (36.5)	1015 (39.6)	343 (13.4)
	Overweight	2287	276 (9.6)	954 (33.0)	1181 (40.9)	476 (16.5)
	Obese	1722	107 (6.2)	550 (31.9)	758 (44.0)	307 (17.8)

*n*(%) unless otherwise stated.

^x^Significant difference between groups (chi-square, *P* < 0.05).

^abc^Significantly higher age compared with other groups with the same subscript (ANOVA and Bonferroni-corrected post-hoc comparisons, *P* < 0.001).

Table 2 shows domain-specific sitting time stratified by unhealthy behaviour score and individual health behaviours. Participants who did not meet the MVPA guidelines sat for 12 minutes/day more at work compared with those who did, these individuals also reported sitting for significantly longer during workday travel and workday/non-workday TV viewing. However, this group had a lower average sitting time during workday leisure-time compared with those who met MVPA guidelines. Smokers reported higher sitting times during TV viewing and when working from home compared with non-smokers. Those exceeding alcohol guidelines reported sitting for an additional 40 minutes/day whilst watching TV compared with those who met the guidelines; these individuals also reported sitting for longer at work, during leisure-time and during non-workday computer-use. Conversely, those who met the alcohol guidelines also sat for longer while travelling on a workday compared with those who did not. Those who met the guidelines for fruit and vegetable intake reported sitting less at work, while TV viewing and during non-workday computer-use compared with those who did not meet the guidelines.

**Table 2:** Domain-specific sitting time (mean ± SD mins/day) on a work and non-workday by unhealthy behaviour classification and score

	*Total sample*	*Physical activity*		*Alcohol consumption*		*Fruit and vegetable consumption*		*Smoking status* *^z^*		*Unhealthy behaviour score*			
	*—*	*Yes*	*No*	*Yes*	*No*	*Yes*	*No*	*Yes*	*No*	*0*	*1*	*2*	*3*
*n* (%)	7170 (100)	1605 (22.4)	5565 (77.6)	5644 (78.7)	1526 (21.3)	3206 (44.7)	3964 (55.3)	866 (12.1)	6304 (87.9)	651 (9.1)	2439 (34.0)	2954 (41.2)	1126 (15.7)
**Workday sitting**													
Travel	79 ± 54	74 ± 57	81 ± 54^x^	81 ± 55^x^	73 ± 53	80 ± 55	79 ± 54	78 ± 60	79 ± 54	79 ± 57	80 ± 55	81 ± 53	75 ± 55
Work	383 ± 95	374 ± 97	386 ± 94^x^	381 ± 96	391 ± 88^x^	380 ± 97	385 ± 93^x^	385 ± 92	383 ± 95	370 ± 101^ab^	381 ± 97^a^	385 ± 93^b^	393 ± 88^a^
TV viewing	94 ± 73	90 ± 70	95 ± 73^x^	89 ± 70	111 ± 80^x^	89 ± 70	97 ± 75^x^	100 ± 79^x^	93 ± 72	81 ± 68^ab^	89 ± 68^a^	93±73^ab^	111 ± 81^a^
Computer use	48 ± 77	48 ± 77	48 ± 77	49 ± 79	47 ± 72	48 ± 79	48 ± 76	44 ± 75	49 ± 78	46 ± 79	50 ± 80	48 ± 76	46 ± 73
Leisure time	39 ± 49	41 ± 50^x^	38 ± 48	38 ± 48	41 ± 53^x^	40 ± 50	38 ± 48	39 ± 52	38 ± 48	42 ± 50	39 ± 48	38 ± 48	39 ± 51
**Non-workday sitting**													
Travel	61 ± 56	61 ± 55	61 ± 56	61 ± 55	59 ± 57	60 ± 54	61 ± 57	57 ± 55	61 ± 56	62 ± 57	60 ± 53	61 ± 57	58 ± 58
Work	72 ± 109	72 ± 106	72 ± 110	71 ± 106	75 ± 119	71 ± 103	73 ± 114	89 ± 124^x^	70 ± 107	69 ± 98	72 ± 104	69 ± 109^a^	82 ± 125^a^
TV viewing	173 ± 101	169 ± 96	174 ± 102^x^	164 ± 96	205 ± 112^x^	161 ± 92	183 ± 107^x^	190 ± 109^x^	170 ± 97	150 ± 86^ab^	161 ± 92^ab^	175 ± 101^ab^	207 ± 117^a^
Computer use	70 ± 69	70 ± 69	70 ± 69	69 ± 67	74 ± 75^x^	66 ± 63	73 ± 73^x^	71 ± 76	69 ± 68	66 ± 64	66 ± 64^a^	72 ± 70	74 ± 78^a^
Leisure time	115 ± 91	116 ± 90	115 ± 91	114 ± 89	121 ± 97^x^	117 ± 91	114 ± 90	119 ± 102	115 ± 89	117 ± 91	113 ± 89	116 ± 90	117 ± 97

^x^Significantly higher sitting time compared with other group (independent *t*-tests, *P* < 0.05).

^ab^Significantly higher sitting time than other groups with the same subscript (ANOVA and Bonferroni-corrected post-hoc comparisons, *P* < 0.001).

^z^No guidelines available for cigarette smoking: ‘Yes’ denotes smokers, ‘No’ denotes non-smokers.

Tables 3 and 4 show the multinomial regression model results exploring the odds of each unhealthy behaviour score associated with low, moderate and high amounts of domain-specific sitting adjusted for BMI, age, sex, marital status, survey year, salary, work pattern and education (see Supplementary Tables 1 and 2 for unadjusted model results). On a workday, no significant associations were found between unhealthy behaviour score and sitting while travelling. Conversely, office workers who sat for ≥6 hours/day at work were more likely to have an unhealthy behaviour score of ≥1 compared with those who sat for ≤6 hours/day. Sitting for ≥7 hours/day at work was associated with double the odds of being in the highest unhealthy behaviour score category compared with those sitting for ≤6 hours. Increased odds were also found for high sitters (≥2 hours) in the workday TV viewing domain who were more likely to have a score of ≥1 and were 119% more likely to be in the highest unhealthy behaviour score category compared with low TV sitters (<1 hour). Conversely, <1 hour of workday computer-use was shown to lower the chances of having an unhealthy behaviour score of 3 by 31% compared with those who did not sit in this domain. Leisure-time sitting was not associated with unhealthy behaviour score.

**Table 3:** Fully adjusted multinomial logistic regression models exploring the association between unhealthy behaviour score and sitting on a workday

*Sitting Time Domain Tertile (mins/day)*		*Unhealthy Behaviour Score (0 = ref, n = 651)*		
		*Fully Adjusted Model* ^*a*^ *OR (95% CI)*		
**Travel**	***n***	**1 (*n* = 2439)**	**2 (*n* = 2954)**	**3 (*n* = 1126)**
Low (0–60)	2173	1.00 (ref)	1.00 (ref)	1.00 (ref)
Moderate (60–90)	2127	1.04 (0.83, 1.31)	1.21 (0.96, 1.51)	1.18 (0.92, 1.53)
High (≥90)	2870	1.02 (0.82, 1.25)	1.08 (0.88, 1.33)	0.89 (0.70, 1.13)
**Work**				
Low (0–360)	1669	1.00 (ref)	1.00 (ref)	1.00 (ref)
Moderate (360–420)	1910	1.41 (1.11, 1.80)^xx^	1.52 (1.20, 1.92)^xx^	1.67 (1.26, 2.21)^xxx^
High (≥420)	3591	1.38 (1.12, 1.71)^xx^	1.62 (1.32, 2.00)^xxx^	2.03 (1.59, 2.61)^xxx^
**TV viewing**				
Low (0–60)	1930	1.00 (ref)	1.00 (ref)	1.00 (ref)
Moderate (60–120)	1867	1.03 (0.82, 1.29)	0.89 (0.71, 1.11)	0.93 (0.71, 1.22)
High (≥120)	3373	1.37 (1.10, 1.70)^xx^	1.48 (1.20, 1.83)^xxx^	2.19 (1.71, 2.80)^xxx^
**Computer use**				
Low (0)	2733	1.00 (ref)	1.00 (ref)	1.00 (ref)
Moderate (1–60)	1846	0.81 (0.65, 1.01)	0.83 (0.67, 1.03)	0.69 (0.54, 0.89)^xx^
High (≥60)	2591	1.14 (0.92, 1.41)	1.05 (0.85, 1.30)	0.92 (0.73, 1.17)
**Leisure-time**				
Low (0)	3298	1.00 (ref)	1.00 (ref)	1.00 (ref)
Moderate (1–60)	2791	0.95 (0.78, 1.15)	0.90 (0.75, 1.09)	0.82 (0.66, 1.02)
High (>60)	1081	0.92 (0.71, 1.19)	0.86 (0.67, 1.11)	0.81 (0.60, 1.08)

^a^Adjusted for BMI, age, sex, marital status, survey year, salary, work pattern and education.

^x^ *P* < 0.05

^xx^ *P* < 0.01

^xxx^ *P* < 0.001

**Table 4:** Fully adjusted multinomial logistic regression models exploring the association between unhealthy behaviour score and sitting on a non-workday

*Sitting Time Domain Tertile (mins/day)*		*Unhealthy Behaviour Score (0 = ref, n = 651)*		
		*Fully Adjusted Model* *^a^* *OR (95% CI)*		
**Travel**	***n***	**1 (*n* = 2439)**	**2 (*n* = 2954)**	**3 (*n* = 1126)**
Low (0–30)	2878	1.00 (ref)	1.00 (ref)	1.00 (ref)
Moderate (30–60)	2541	0.93 (0.76, 1.14)	1.00 (0.82, 1.22)	0.78 (0.62, 0.99)^x^
High (>60)	1751	0.91 (0.72, 1.13)	0.93 (0.74, 1.16)	0.75 (0.58, 0.97)^x^
**Work**				
Low (0)	4284	1.00 (ref)	1.00 (ref)	1.00 (ref)
Moderate (1–180)	1366	0.98 (0.79, 1.23)	0.81 (0.65, 1.01)	0.61 (0.47, 0.81)^xxx^
High (≥180)	1520	1.01 (0.80, 1.26)	0.91 (0.73, 1.14)	1.15 (0.90, 1.48)
**TV viewing**				
Low (0–120)	3073	1.00 (ref)	1.00 (ref)	1.00 (ref)
Moderate (121–180)	1783	1.32 (1.06, 1.64)^x^	1.44 (1.16, 1.78)^xx^	1.76 (1.37, 2.28)^xxx^
High (≥180)	2314	1.38 (1.11, 1.72)^xx^	1.82 (1.47, 2.26)^xxx^	2.96 (2.32, 3.77)^xxx^
**Computer use**				
Low (0–30)	1680	1.00 (ref)	1.00 (ref)	1.00 (ref)
Moderate (30– 60)	3269	1.04 (0.83, 1.29)	1.05 (0.84, 1.31)	0.85 (0.66, 1.09)
High (≥60)	2221	1.14 (0.89, 1.45)	1.21 (0.95, 1.54)	0.95 (0.72, 1.25)
**Leisure-time**				
Low (0–60)	2916	1.00 (ref)	1.00 (ref)	1.00 (ref)
Moderate (60–120)	2141	0.99 (0.80, 1.22)	1.09 (0.88, 1.34)	0.98 (0.97, 1.25)
High (≥121)	2113	0.91 (0.73, 1.12)	0.98 (0.79, 1.21)	0.92 (0.73, 1.17)

^a^Adjusted for BMI, age, sex, marital status, survey year, salary, work pattern and education.

^x^ *P* < 0.05

^xx^ *P* < 0.01

^xxx^ *P* < 0.005

On a non-workday, sitting while travelling for ≥30 minutes/day was associated with a 25% reduction in the odds of having an unhealthy behaviour score of 3 compared with ≤30 minutes/day. Office workers who reported sitting for ≤2 hours while working at home were 40% less likely to have an unhealthy behaviour score of 3 compared with those who did not sit in this domain. Sitting for 2–3 hours and ≥3 hours/day on a non-workday while watching TV had a 76 and 196% increase in the odds of being in the highest unhealthy behaviour score category compared with those who reported sitting for <2 hours. No significant associations were found between unhealthy behaviour score and computer-use or leisure-time sitting.

## Discussion

### Main finding of this study

This study aimed to explore the association between domain-specific sitting time and other health behaviours including physical activity, alcohol consumption, cigarette smoking and fruit and vegetable intake in a sample of office workers. Sitting for ≥7 hours at work and ≥2 hours while watching TV on a workday both more than doubled the odds of partaking in ≥3 other unhealthy behaviours and sitting while watching TV on a non-workday for 3 hours nearly tripled the odds independent of confounding variables. Conversely, participants in the moderate sitting time category for workday computer-use and working at home as well as ≥30 minutes of non-workday sitting while travelling were associated with lower odds of having ≥3 other unhealthy behaviours. However, the magnitude was small (OR ≥ 0.61) and negligible differences were observed between the highest and lowest unhealthy behaviour score groups.

### What is already known on this topic

No previous studies have examined the association between domain-specific sitting time and multiple other unhealthy behaviours. However, previous research into the associations between sitting time and individual health behaviours supports the current study. Data from the Australian Diabetes, Obesity and Lifestyle (AusDiab) study found that each 30-minute increase in leisure-time physical activity per week was associated with a small significant decrease in the odds of men being in the highest occupational sitting group. Additionally, it was observed that men and women who had low levels of occupational sitting were more likely to be active in their leisure-time.^31^ This supports the findings of the current study where physically inactive office workers were significantly more sedentary at work compared with their active counterparts. The lack of association between smoking and sitting at work found in the current study is supported by Tissot *et al.*,^32^ who analyzed a survey of Quebec employees and found smoking did not influence workplace sitting time.

No previous study has looked at sitting at work and alcohol intake specifically, but Uijtdewilligen and colleagues^33^ found that among Australians, high-risk alcohol drinkers sat for significantly longer than low-risk drinkers on a weekday. This supports the current study where alcohol overconsumption was associated with increased sitting at work. However, the comparison is limited due to the Australian study measuring sitting time across the whole weekday. Similarly, no study has explored fruit and vegetable intake in relation to sitting at work where a negative association was found in the current study, although one study found a positive association between energy intake and occupational sitting in men from the AusDiab study.^31^ High occupational sitters have also been shown to sit for longer outside of work compared with low occupational sitters which could further explain the positive association between the work domain and unhealthy behaviour score.^34^

The negative associations between sitting while TV viewing and individual health behaviours found in this study have been reported elsewhere. Hamer *et al.*^35^ analyzed 4000 adults from the 2003 Scottish Health Survey and found an inverse trend for physical activity and fruit and vegetable intake with those meeting the guidelines sitting less while watching TV or screen-based entertainment. Potential mechanisms for this could be that TV viewing displaces time spent in MVPA^36^ and is associated with increased unhealthy food and beverage consumption^37^ which could displace fruit and vegetable consumption. An increase in smoking has also been linked to TV advertisements^38^ and Hamer *et al.*^35^ found that smokers reported sitting for longer while TV viewing than non-smokers. The current study is further supported by Pereira and colleagues^39^ who found that TV viewing time was positively associated with smoking and low fruit consumption in a sample from the 1958 British birth cohort.

### What this study adds

This is the first study to explore domain-specific sitting time in relation to multiple health behaviours. High amounts of sitting at work and during TV viewing on a work and non-workday are associated with partaking in multiple unhealthy behaviours. This study highlights the importance of sedentary behaviour as it is highly prevalent and associated with current ‘SNAP’ health behaviours, thus should be considered as part of these lifestyle measures in research and health practice. Multicomponent interventions have shown reductions in sedentary time at work using active workstations and additional strategies^40^ but have not targeted or measured the effect on sedentary time while watching TV.

Future interventions should consider sedentary behaviours both at work and during TV viewing to measure the impact on health and other health behaviours. The current study explored the associations between cigarette smoking and domain-specific sitting time; however, with the increase in e-cigarette use, future studies should examine all forms of smoking in relation to sitting time. Interventions are needed to target reducing sitting time in the workplace and TV viewing domains in addition to improving other health behaviours, including smoking, alcohol, physical activity and fruit and vegetable intake. Further research is needed to establish the direction of causation in these associations, and public health policy should consider sitting time as an important health behaviour.

### Limitations of this study

The cross-sectional design does not allow for causality to be established thus it is unclear whether high domain-specific sitting time is a result of a high unhealthy behaviour score or the reverse. This information is needed to inform future interventions targeting a reduction in unhealthy behaviours. Additionally, the combination of unhealthy behaviours within each score could vary and should be taken into consideration when interpreting the results. Conversely, this is the first study to explore this relationship, highlighting the association and warranting further research. The large confidence intervals in some sitting time domains are possibly due to the self-report measure and introduction of recall bias. This could be partly explained by the fact that sedentary behaviours often occur simultaneously increasing recall difficulty.^41^ Additionally, time spent cycling could have been reported by office workers in the travel domain which would confound the results as cycling is beneficial to health. Objective measures have higher validity and measure how sitting time is accumulated but cannot provide context which is a strength of this study as it has identified key domains for interventions.

The other health behaviours were also self-reported and could be subject to biases, but this method allowed for a large sample to be obtained and the information was dichotomized reducing the influence of biases. We cannot, however, rule out the possibility of residual confounding. Other unadjusted confounding factors such as urbanization, well-being and quality of life associated with sitting time could have contributed to the results.^42^ The survey had a low response rate which could influence the representativeness of the sample and inference of results to the wider population. However, similar response rates are common in workplace wellness studies^43^ with non-responders usually having lower socio-economic status thus worse lifestyles. Therefore, it is likely that the results of this study provide a conservative estimate. Furthermore, a large sample was obtained and the average sitting time was similar to previous office worker studies.^44^

## Supplementary Material

Stormont_manuscript-final_supplementary_tables_fdab298

## Notes

**Victoria E. Kettle**, Research Associate

**Mark Hamer**, Professor

**Fehmidah Munir**, Professor

**Jonathan Houdmont**, Assistant Professor

**Kelly Wilson**, PhD candidate

**Robert Kerr**, Senior Lecturer

**Ken Addley**, Professor

**Lauren B. Sherar**, Professor

**Stacy A. Clemes**, Reader
