# Supplementary material for: Cross-sectional associations between domain-specific sitting time and other lifestyle health behaviours: the Stormont study
Source: J Public Health (Oxf). 2021 Aug 3;44(1):51–9. doi: 10.1093/pubmed/fdab298 (PMC8904248; doi:10.1093/pubmed/fdab298)
Supplement: Stormont_manuscript-final_supplementary_tables_fdab298 [file stormont_manuscript-final_supplementary_tables_fdab298.docx]

**Supplementary Table 1** Unadjusted multinomial logistic regression models exploring the association between unhealthy behaviour score and sitting on a workday

| **Sitting Time Domain Tertile (mins/day)** | | **Unhealthy Behaviour Score (0 = ref, n=651)** | | |
| --- | --- | --- | --- | --- |
|  |  | **Unadjusted Model OR (95% CI)** | | |
| **Travel** | **n** | **1 (n=2439)** | **2 (n=2954)** | **3 (n=1126)** |
| Low (0-<60) | 2173 | 1.00 (ref) | 1.00 (ref) | 1.00 (ref) |
| Moderate (60-<90) | 2127 | 1.07 (0.86, 1.34) | 1.25 (1.00, 1.55) | 1.17 (0.91, 1.50) |
| High (≥90) | 2870 | 1.04 (0.85, 1.28) | 1.12 (0.92, 1.37) | 0.90 (0.72, 1.14) |
| **Work** |  |  |  |  |
| Low (0-<360) | 1669 | 1.00 (ref) | 1.00 (ref) | 1.00 (ref) |
| Moderate (360-<420) | 1910 | 1.41 (1.11, 1.78)^xx^ | 1.48 (1.18, 1.87)^xx^ | 1.64 (1.25, 2.15)^xxx^ |
| High (≥420) | 3591 | 1.42 (1.15, 1.74)^xx^ | 1.62 (1.33, 1.99)^xxx^ | 1.96 (1.55, 2.48)^xxx^ |
| **TV viewing** |  |  |  |  |
| Low (0-<60) | 1930 | 1.00 (ref) | 1.00 (ref) | 1.00 (ref) |
| Moderate (60-<120) | 1867 | 1.03 (0.83, 1.29) | 0.86 (0.69, 1.08) | 0.83 (0.64, 1.08) |
| High (≥120) | 3373 | 1.32 (1.07, 1.62)^x^ | 1.40 (1.14, 1.72)^xx^ | 2.07 (1.64, 2.63)^xxx^ |
| **Computer use** |  |  |  |  |
| Low (0) | 2733 | 1.00 (ref) | 1.00 (ref) | 1.00 (ref) |
| Moderate (1-<60) | 1846 | 0.81 (0.66, 1.01) | 0.81 (0.65, 0.99)^x^ | 0.62 (0.49, 0.79)^xxx^ |
| High (≥60) | 2591 | 1.09 (0.89, 1.34) | 1.05 (0.86, 1.29) | 0.98 (0.78, 1.22) |
| **Leisure-time** |  |  |  |  |
| Low (0) | 3298 | 1.00 (ref) | 1.00 (ref) | 1.00 (ref) |
| Moderate (1–60) | 2791 | 0.93 (0.77, 1.12) | 0.88 (0.73, 1.06) | 0.81 (0.66, 1.00) |
| High (>60) | 1081 | 0.88 (0.69, 1.14) | 0.84 (0.65, 1.07) | 0.84 (0.63, 1.11) |

^x^p<0.05

^xx^p<0.01

^xxx^p<0.001

**Supplementary Table 2** Unadjusted multinomial logistic regression models exploring the association between unhealthy behaviour score and sitting on a non-workday

| **Sitting Time Domain Tertile (mins/day)** | | **Unhealthy Behaviour Score (0 = ref, n=651)** | | |
| --- | --- | --- | --- | --- |
|  |  | **Unadjusted Model OR (95% CI)** | | |
| **Travel** | **n** | **1 (n=2439)** | **2 (n=2954)** | **3 (n=1126)** |
| Low (0 - <30) | 2878 | 1.00 (ref) | 1.00 (ref) | 1.00 (ref) |
| Moderate (30 - <60) | 2541 | 0.91 (0.75, 1.11) | 0.98 (0.80, 1.19) | 0.75 (0.60, 0.94)^x^ |
| High (>60) | 1751 | 0.89 (0.72, 1.11) | 0.90 (0.72, 1.12) | 0.74 (0.57, 0.94)^x^ |
| **Work** |  |  |  |  |
| Low (0) | 4284 | 1.00 (ref) | 1.00 (ref) | 1.00 (ref) |
| Moderate (1 - <180) | 1366 | 0.94 (0.76, 1.17) | 0.74 (0.60, 0.92)^xx^ | 0.52 (0.40, 0.67)^xxx^ |
| High (≥180) | 1520 | 0.98 (0.78, 1.22) | 0.89 (0.71, 1.03) | 1.14 (0.90, 1.45) |
| **TV viewing** |  |  |  |  |
| Low (0 - <120) | 3073 | 1.00 (ref) | 1.00 (ref) | 1.00 (ref) |
| Moderate (121 - <180) | 1783 | 1.27 (1.02, 1.57)^x^ | 1.37 (1.11, 1.70)^xx^ | 1.71 (1.34, 2.20)^xxx^ |
| High (≥180) | 2314 | 1.30 (1.05, 1.61)^x^ | 1.79 (1.45, 2.20)^xxx^ | 3.32 (2.63, 4.20)^xxx^ |
| **Computer use** |  |  |  |  |
| Low (0 – <30) | 1680 | 1.00 (ref) | 1.00 (ref) | 1.00 (ref) |
| Moderate (30 – <60) | 3269 | 1.06 (0.85, 1.32) | 1.07 (0.86, 1.32) | 0.82 (0.64, 1.04) |
| High (≥60) | 2221 | 1.09 (0.86, 1.38) | 1.21 (0.96, 1.53) | 1.07 (0.83, 1.39) |
| **Leisure-time** |  |  |  |  |
| Low (0 – <60) | 2916 | 1.00 (ref) | 1.00 (ref) | 1.00 (ref) |
| Moderate (60 – <120) | 2141 | 0.99 (0.80, 1.22) | 1.07 (0.87, 1.32) | 0.95 (0.75, 1.20) |
| High (≥121) | 2113 | 0.91 (0.74, 1.12) | 0.98 (0.80, 1.20) | 0.95 (0.75, 1.19) |

^x^p<0.05

^xx^p<0.01

^xxx^p<0.005
